# Editorial: Developmental, Modal, and Pathological Variation—Linguistic and Cognitive Profiles for Speakers of Linguistically Proximal Languages and Varieties

**DOI:** 10.3389/fpsyg.2018.01804

**Published:** 2018-09-24

**Authors:** Kleanthes K. Grohmann, Maria Kambanaros, Evelina Leivada

**Affiliations:** ^1^Department of English Studies, University of Cyprus, Nicosia, Cyprus; ^2^Cyprus Acquisition Team, CAT Lab, Nicosia, Cyprus; ^3^Department of Rehabilitation Sciences, Cyprus University of Technology, Limassol, Cyprus; ^4^Department of Language and Culture, UiT–The Arctic University of Norway, Tromsø, Norway

**Keywords:** (a)typical development, cognitive advantage, multilectalism, proximity, varieties

One significant area of research in the multifaceted field of bilingualism over the past two decades, spanning among many others from Green (1998) to Chung-Fat-Yim et al. (2016), has been the demonstration, validation, and account of the so-called “bilingual advantage.” This refers to the hypothesis that bilingual speakers have advanced abilities in executive functions (EF) and other domains of human cognition. Such cognitive benefits of bilingualism have an impact on the processing mechanisms active during language acquisition in a way that results in language variation. Within bilingual populations, the notion of language proximity (or linguistic distance) is also of key importance for deriving variation. In addition, sociolinguistic factors can invest the process of language development and its outcome with an additional layer of complexity, such as schooling, language, dominance, competing motivations, or the emergence of mesolectal varieties, which blur the boundaries of grammatical variants. This is particular relevant for diglossic speech communities-bilectal, bidialectal, or bivarietal speakers.

The defined goal of the present Research Topic is to address whether the bilingual advantage extends to such speakers as well. Thus, “Linguistic and Cognitive Profiles for Speakers of Linguistically Proximal Languages and Varieties” become an important matter within “Developmental, Modal, and Pathological Variation.” The larger issue of cognitive-linguistic representations in bilingual speakers is expressed in Putnam et al.'s model for determining language proximity. Building on Hsin's (2014) Integration Hypothesis, the authors sketch a framework in which “bilingual grammars are neither isolated, nor (completely) conjoined with one another in the bilingual mind, but rather exist as integrated source grammars that are further mitigated by a common, combined grammar.” Once linguistic distance between the languages of bilingual speakers is measured in computational cognitive architectures, any effects of a bilingual advantage in terms of cognition and memory can be assessed empirically. One such empirical assessment is presented by Bosma et al. who investigate whether degree of bilingualism in Frisian-Dutch children influences EF—and if so, whether this effect is sustained over time. To this effect, they analyzed longitudinal data from Frisian-Dutch bilingual children. The results confirm that “cognitive effects of bilingualism are moderated by degree of bilingualism,” where the amount of exposure in the minority language (i.e., m the home variety) indirectly affects bilingual children's cognitive development. However, as the authors stress, “the findings also demonstrate that the effect of bilingualism on EF is limited and unstable”—a take-home message that is in line with what recent reviews have suggested in relation to the bilingual advantage (Paap et al., 2014; Lehtonen et al., 2018).

A set of three papers further investigates the purported bilingual advantage in combination with sociolinguistic and socio-economic considerations. Blom et al. tested whether the sociolinguistic context of language use affects the bilingual advantage. And indeed, bilingual children outperformed their monolingual peers on selective attention, presumably because they focused on different aspects of the task. Garraffa et al. explore “the effects of bilingualism in Sardinian as a regional minority language on the linguistic competence in Italian as the dominant language and on non-linguistic cognitive abilities” with adults living in Sardinia. No evidence for a “bilingual advantage” emerged through the task that tapped into the cognitive control of attention, but bilinguals did perform better than monolinguals on working memory tasks. In addition, “[b]ilinguals with lower formal education were found to be faster at comprehension of one type of complex sentence,” while “bilinguals and monolinguals with higher education showed comparable slower processing of complex sentences.” Meir and Armon-Lotem explore the influence of socioeconomic status (SES) and bilingualism on the linguistic skills and verbal short-term memory of Hebrew-Russian bilingual preschoolers, half from low SES backgrounds. The authors propose that bilingualism is associated with decreased vocabulary size and lower performance on verbal short-term memory tasks, while SES also impacts verbal short-term memory with lowest linguistic load. They also argue that “an unprivileged background has a negative impact on children's cognitive development.”

Effects of language or linguistic proximity, bi-/multilectal acquisition, and their relevance for the socio-syntax of language development are of particular interest to this Research Topic—that is, apparent sociolinguistic aspects such as formal schooling that may have an effect on the grammatical language development of a child growing up in a bi- or multilectal society. Considering the case of Brazilian (L1) and European (L2) Portuguese bidialectal adults that had moved to Portugal as adults, Castro et al. explore possible differences in the interpretation of null and overt object pronouns. They “test the extent to which […] speakers display cross-linguistic influence in either direction.” The high degree of typological proximity between the speakers' linguistic varieties is argued to contribute to L1 attrition and hinder target-like L2 performance at the same time.

There are also four contributions that focus on the differences between the two varieties of Greek spoken in Cyprus. When asked to make acceptability judgments, the performance of speakers of non-standard varieties may actually be subject to interference from factors such as prescriptive notions of grammatical correctness and sociolinguistic values typically attached to “dialects.” Recognizing the importance of working with corpora of spontaneous speech, Leivada et al. investigate variation in the spontaneous productions of adult speakers of the non-standard variety Cypriot Greek. In their corpus, they observed intraspeaker realizations of different values of the same variant within the same syntactic environment; a result that is incompatible with the mainstream “triggering-a-single-value” approach of parametric models. Since the analysis of these conflicting values is ultimately a way of investigating Universal Grammar primitives, the authors further conclude that claims about the alleged unfalsifiability of Universal Grammar are empirically unfounded. Tsiplakou explores the concept of gradient bilectalism by capitalizing on insights from recent developments in second language acquisition, particularly the suggestion that aspects of the syntax-discourse interface that are not easily accessible to the learner may lead to fossilization, even at end state. Based on quantitative data from a questionnaire survey, she suggests that imperfect acquisition of some structural aspects of the standard language may affect bilectals' performance in a way that involves a transfer of features from the dialect to the standard. Themistocleous investigates the effects of two linguistically proximal Modern Greek dialects, Athenian Greek and Cypriot Greek on the temporal, spectral, and co-articulatory properties of fricatives with the aim to determine the acoustic properties that convey information about these two dialects. The results revealed that Athenian Greek and Cypriot Greek fricatives differ in all spectral properties across all places of articulation. The co-articulatory effects of fricatives on following vowel were different across the two varieties, something that suggests that dialectal information is encoded in the acoustic structure of fricatives. The contribution by Ayiomamitou and Yiakoumetti deals with regional linguistic variation and its implications for education by focusing on the Greek Cypriot educational context. The aim of the study was to understand Greek Cypriot primary school pupils' sociolinguistic awareness via examination of their written production in their home variety. The students were advised to produce texts that reflected their everyday way of talking with family and friends (beyond school boundaries and the formal register this environment may induce). The authors found students' texts to include many mesolectal features but also “a significant and unexpected number of basilectal features and instances of hyperdialectism,” which rendered their texts register-inappropriate.

Merging sociolinguistic and neurocognitive insights about language variation, three papers seek to uncover which factors derive variation in the course of language development, that is, how variation in cases of pathological development affects different parts of language and whether the affected markers are manifested in a comparable way. For starters, it is common to find that “minority” languages enjoy fewer (if any) diagnostic tools than “majority” languages. This has repercussions for the detection and proper assessment of children with Specific Language Impairment (SLI) brought up in these languages. With a view to remedy this situation for Catalan, Gavarró developed a sentence repetition task to assess grammatical maturity in school-age children. The findings display clear differences with typically developing children providing identical repetition at twice the rate of children with SLI. Moreover, the children with SLI had more deviant productions, both ungrammatical ones and grammatical yet different repetitions. Saiegh-Haddad and Ghawi-Dakwar tested phonological and lexical distance between a dialect of Palestinian Arabic spoken in the north of Israel and Modern Standard Arabic on word and non-word repetition in children with SLI and age-matched controls. The authors find that children with SLI underperform on all tasks and point to a “general phonological memory deficit.” They also argue that the results “reflect the role of linguistic distance in phonological memory for novel linguistic units in Arabic SLI,” which in turn would “support a specific Linguistic Distance Hypothesis of SLI in a diglossic setting.” Previous work on linguistic abilities of individuals with Down syndrome (DS) suggests severe impairment of complex syntactic structures in a number of languages. Given difficulties reported with comprehension and production of relative clauses and object clitics in typically developing Greek Cypriot bilectal children, one could hypothesize that the bilectal environment in which children with DS grow up may cause an added difficulty in the acquisition of other complex syntactic structures, such that of the understudied syntactically complex subjunctives. Christodoulou and Grohmann examine whether Greek Cypriot bilectal children and adolescents with DS evidence an impairment with the comprehension of subjunctive clauses, corroborating arguments for an overall syntactic impairment from past research on DS. Full analysis of the comprehension data evidenced high means of accuracy, with parallel performance across the two groups. The linguistic differences between Cypriot and Standard Modern Greek do not appear to affect the acquisition of subjunctives.

As its title suggests, the Research Topic “Developmental, Modal, and Pathological Variation—Linguistic and Cognitive Profiles for Speakers of Linguistically Proximal Languages and Varieties” aimed to approach the topic of language variation from different perspectives. To this end, we brought together studies on typologically different languages (both standard and non-standard), ranging from infancy into adulthood, for speakers with different cognitive phenotypes as well as different language backgrounds (e.g., heritage languages in diaspora). The contributions to this research topic are informative with respect to certain key aspects within current linguistic research such as the bilingual advantage, the passive knowledge of the standard in bi(dia)lectal speakers, aspects of transfer, and the key role of SES in cognitive and linguistic development. As Noam Chomsky has repeatedly argued, in order to understand the human capacity to acquire and use language, we need to know *what options it permits* (Chomsky, 2015) through studying language variation, and this Research Topic aims to take a multidisciplinary step into this direction.

## Author contributions

KG wrote a first draft of this editorial, which EL completed and MK edited further. All authors listed have made a substantial, direct and intellectual contribution to the work, and approved it for publication.

### Conflict of interest statement

The authors declare that the research was conducted in the absence of any commercial or financial relationships that could be construed as a potential conflict of interest.
